# Efficient Generation of Integration-Free iPS Cells from Human Adult Peripheral Blood Using BCL-XL Together with Yamanaka Factors

**DOI:** 10.1371/journal.pone.0064496

**Published:** 2013-05-21

**Authors:** Rui-Jun Su, David J. Baylink, Amanda Neises, Jason B. Kiroyan, Xianmei Meng, Kimberly J. Payne, Benjamin Tschudy-Seney, Yuyou Duan, Nancy Appleby, Mary Kearns-Jonker, Daila S. Gridley, Jun Wang, K-H. William Lau, Xiao-Bing Zhang

**Affiliations:** 1 Department of Medicine, Loma Linda University, Loma Linda, California, United States of America; 2 Division of Anatomy, Loma Linda University, Loma Linda, California, United States of America; 3 Center for Health Disparities and Molecular Medicine, Loma Linda University, Loma Linda, California, United States of America; 4 Department of Internal Medicine, Institute for Regenerative Cures, University of California Davis Medical Center, Sacramento, California, United States of America; 5 Department of Radiation Medicine, Loma Linda University, Loma Linda, California, United States of America; 6 Department of Pathology, Loma Linda University, Loma Linda, California, United States of America; 7 Jerry L. Pettis Memorial VA Medical Center, Loma Linda, California, United States of America; French Blood Institute, France

## Abstract

The ability to efficiently generate integration-free induced pluripotent stem cells (iPSCs) from the most readily available source—peripheral blood—has the potential to expedite the advances of iPSC-based therapies. We have successfully generated integration-free iPSCs from cord blood (CB) CD34^+^ cells with improved oriP/EBNA1-based episomal vectors (EV) using a strong spleen focus forming virus (SFFV) long terminal repeat (LTR) promoter. Here we show that Yamanaka factors (OCT4, SOX2, MYC, and KLF4)-expressing EV can also reprogram adult peripheral blood mononuclear cells (PBMNCs) into pluripotency, yet at a very low efficiency. We found that inclusion of BCL-XL increases the reprogramming efficiency by approximately 10-fold. Furthermore, culture of CD3^−^/CD19^−^ cells or T/B cell-depleted MNCs for 4–6 days led to the generation of 20–30 iPSC colonies from 1 ml PB, an efficiency that is substantially higher than previously reported. PB iPSCs express pluripotency markers, form teratomas, and can be induced to differentiate in vitro into mesenchymal stem cells, cardiomyocytes, and hepatocytes. Used together, our optimized factor combination and reprogramming strategy lead to efficient generation of integration-free iPSCs from adult PB. This discovery has potential applications in iPSC banking, disease modeling and regenerative medicine.

## Introduction

The successful generation of induced pluripotent stem cells (iPSCs) from human somatic cells has revolutionized our understanding of the development and regeneration of cells, tissues and organs, igniting new hope for replacement therapies.[1], [2], [3] This breakthrough has been recently recognized by the Nobel Committee for Physiology or Medicine.[4], [5], [6] The development of novel approaches for generating integration-free iPSCs has eliminated the concern of integrating virus-associated genotoxicity in clinical applications. Transposon [7], [8] and excisable polycistronic lentiviral vectors [9], [10], [11], [12], [13], [14] can be used to generate integration-free iPSCs, but a second step is necessary to remove the transgenes once reprogramming has been achieved. Many one-step approaches such as adenovirus vectors, [15], [16] plasmids, [17], [18], [19] minicircle DNAs, [20], [21] artificial chromosome vectors [22] and protein transduction [23], [24] are very inefficient in generating integration-free iPSCs. Relatively efficient approaches that have been readily reproduced in different labs include Sendai virus vector, [25], [26], [27], [28], [29], [30] modified mRNA, [31], [32], [33] and oriP/EBNA1-based episomal vectors (EV). [34], [35], [36], [37], [38], [39], [40], [41], [42] The most cost effective approach is EV, because there is no need for packaging of viral vectors and one simple infection instead of daily or multiple additions of factors is sufficient for successful reprogramming. EV is a plasmid containing two elements from Epstein-Bar virus: oriP and EBNA1. Binding of the EBNA1 protein to the virus replicon region oriP maintains a relatively long-term episomal presence of plasmids in mammalian cells. The unique features of EV make it an ideal vector for generating integration-free iPSCs. EV yields expression of reprogramming factors at sufficiently high levels for several cell divisions, thus allowing for successful reprogramming after only one infection, while the gradual depletion of plasmids during each cell division leads to the generation of integration-free iPSCs after approximately 2 months of culture.

Although fibroblasts from skin biopsy or other sources were initially used in many studies for the generation of iPSCs, mononuclear cells (MNCs) from peripheral blood (PB) have been widely accepted as a more convenient and almost unlimited resource for cell reprogramming. [43], [44], [45], [46] PB MNCs are a mixed population, containing lymphoid cells like T cells and B cells and non-lymphoid cells that include myeloid cells as well as 0.01–0.1% CD34^+^ hematopoietic stem/progenitor cells (HSCs). In earlier studies, mature T or B cells were efficiently converted to iPSCs with Sendai virus or EV plasmids. [25], [42], [47] However, iPSCs generated from T/B cells contain T cell receptor (TCR) or immunoglobulin (IG) gene rearrangements, restricting their broad applications in regenerative medicine. [45], [46], [48] Therefore, we and many other investigators have attempted to generate integration-free iPSCs from non-lymphoid cells. [35], [39], [40], [41] However, only 1–5 integration-free iPSC colonies can be generated from 1 ml of PB in these reports. Thus, further improvements in reprogramming efficiency are necessary to make the EV-based approach for reprogramming PB widely applicable. Building on our previous finding that our improved EV vector design leads to efficient reprogramming of cord blood (CB) CD34^+^ cells, [40] here we further develop this approach for the generation of integration-free iPSCs from adult PBMNCs. More recent approaches generate up to 10 iPSC colonies from 1 ml of PB in non-T cell culture conditions with 7 factors including EBNA1 and shRNA against TP53 (also known as p53). [42] However, expression of EBNA1 and TP53 shRNA synergistically inhibits the genome guardian p53, which raises concerns about the genomic integrity of iPSCs generated using this approach. [49] Thus, we avoided the use of TP53 shRNA in this study.

## Results

### BCL-XL enhances the efficiency of OS-mediated reprogramming of both CB CD34^+^ cells and PBMNCs

We have found that balanced expression of OCT4 and SOX2 (OS) driven by a single strong promoter efficiently induces CB CD34^+^ cells into pluripotency. [40] It was also reported that Bcl2 increases reprogramming efficiency of mouse fibroblasts by 2–3 fold. [50] We thus hypothesized that anti-apoptotic factors in the Bcl-2 family may increase OS-mediated human blood cell reprogramming. To test this hypothesis, we cloned anti-apoptotic factors BCL2, BCL-XL (isoform Bcl-X(L) of BCL2L1), and MCL1 into a lentiviral vector under the control of the spleen focus forming virus (SFFV) promoter. We chose this promoter because we and others found that the SFFV promoter drives higher-levels of transgene expression in primary hematopoietic cells or cell lines than commonly used promoters like human elongation factor 1alpha (EF1), human phosphoglycerate kinase (PGK), and cytomegalovirus (CMV). [40], [51], [52]

**Figure 1:**
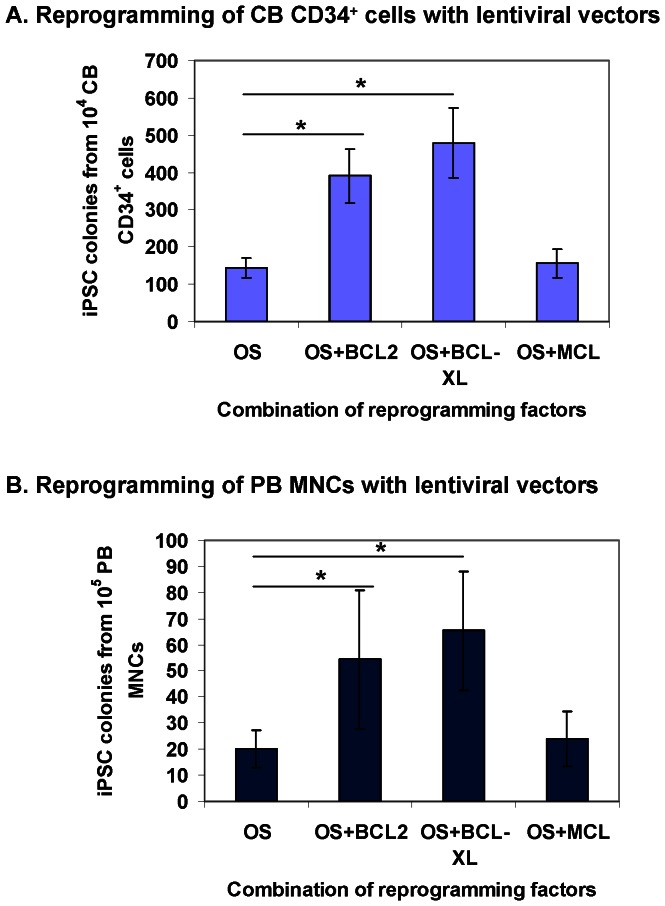
BCL-XL significantly enhances OS-mediated reprogramming of cord blood and peripheral blood cells with lentiviral vectors. (**A**) Differential effects of BCL2 family members on enhancing OS-mediated reprogramming of CB cells. CB CD34^+^ cells were cultured for 2 days before lentiviral transduction. CB iPSC colonies were enumerated at 2 weeks after transduction of reprogramming factors. Data shown are presented as mean ± SEM (n = 4). OS: OCT4 and SOX2. * indicates *P*<0.05. (**B**) Differential effects of BCL2 family members on enhancing OS-mediated reprogramming of PBMNCs. PBMNCs were cultured for 4–6 days before lentiviral transduction. PB iPSC colonies were enumerated at 3 weeks after transduction of reprogramming factors. Data shown are presented as mean ± SEM (n = 4). OS: OCT4 and SOX2. PBMNCs, peripheral blood mononuclear cells. * indicates *P*<0.05. BCL2 and BCL-XL significantly increased reprogramming of both CB CD34^+^ cells and PBMNCs.

Consistent with our previous report, [40] 1–2% of OS-transduced CB CD34^+^ cells were converted to iPSCs (**Figure 1A**). Inclusion of BCL2 or BCL-XL increased reprogramming efficiency by ∼3-fold (*P* < 0.05), while MCL1 had no obvious effect on enhancing OS-mediated reprogramming. We then conducted the same study using adult PB. MNC were isolated from several donors (age 22–43) by Ficoll-Hypaque density gradient centrifugation and cultured for 4–6 days. Of note, OS alone could also induce adult blood cells into pluripotency, although the efficiency was 100-fold lower than reprogramming of CB CD34^+^ cells (**Figure 1B**). Of interest, the effects of the three anti-apoptotic factors on OS-mediated reprogramming of PBMNCs were identical to that of CB CD34^+^ cells (**Figure 1B**). BCL-XL appeared to be more potent than BCL2 in enhancing reprogramming, even though the difference did not reach statistical significance, Therefore, we elected BCL-XL in our integration-free iPSC studies.

We have shown that our lentiviral vector design enables high-level expression of reprogramming factors, leading to a 1000-fold increase in reprogramming of CB CD34^+^ cells relative to previously reported. [40], [53] To investigate if this conclusion also holds for PB reprogramming, we compared our results with a similar study reported by Daley's lab. [46] They generated iPSCs from human PBMNCs at an efficiency of up to 0.001% with four factors (OS + MYC and KLF4 or MK) expressed by lentiviral vectors. The reprogramming efficiency with OS alone was 0.02% (**Figure 1B**); with the addition of MK, 0.2% PBMNCs can be reprogrammed into iPSCs. Addition of MK to OS increased PBMNC reprogramming efficiency by 10-fold, which is similar to our results with CB CD34^+^ cells. [54] These data suggest that our lentiviral vector-mediated PBMNC reprogramming is at least 200-fold more efficient than other vectors. [46]

### Generation of integration-free iPSCs from PBMNCs with episomal vectors

**Figure 2:**
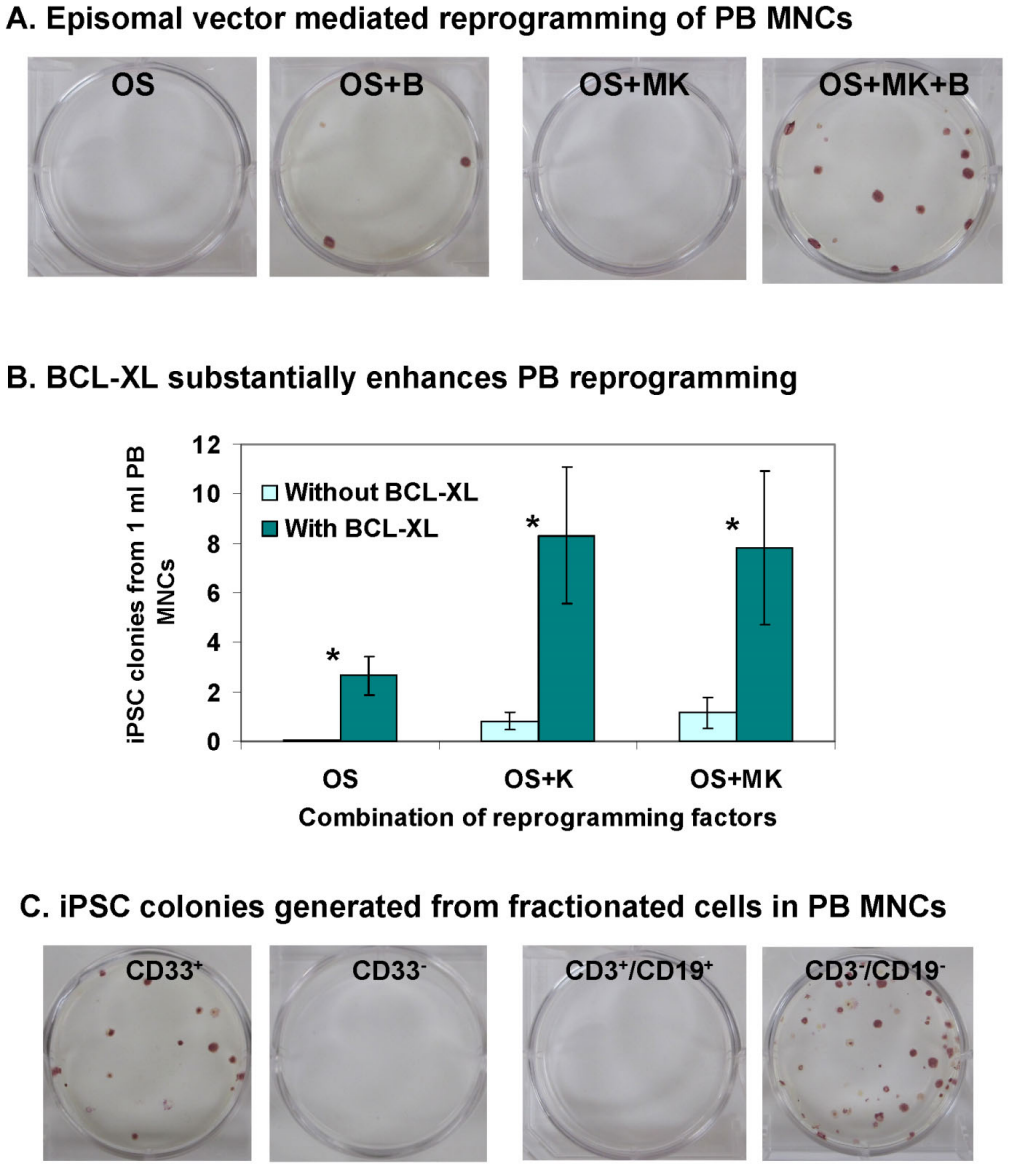
Generation of integration-free iPSCs from adult PBMNCs with episomal vectors. (**A**) ALP staining of iPSCs at 4 weeks after nucleofection of PBMNCs with reprogramming factor-expressing episomal vectors. OS, OCT4 and SOX2; MK, MYC and KLF4; B, BCL-XL. PBMNCs were cultured for 4–8 days before nucleofection. 1×10^6^ PBMNCs were nucleofected and then seeded into each well. (**B**) Inclusion of BCL-XL increases PB reprogramming efficiency by up to 10-fold. PBMNCs were cultured for 4–8 days before nucleofection. ALP-positive iPSC colonies were enumerated at 3–4 weeks after nucleofection. Data are presented as mean ± SEM (n = 6). In all 3 conditions, BCL-XL significantly increased reprogramming efficiency. * indicates *P*<0.05. (**C**) iPSC are generated from PBMNCs expressing the myeloid lineage marker, CD33, but not lymphoid cells (CD3^+^ and CD19^+^ cells) in PBMNCs. ALP staining of iPSCs at 4 weeks after nucleofection of fractionated PBMNCs with episomal vectors OS+MK+B. CD33, myeloid marker; CD3, T cell marker; CD19, B cell marker. 1×10^6^ indicated cells were nucleofected and then seeded into each well. ALP staining was conducted at 4 weeks after nucleofection.

We have developed an improved oriP/EBNA-based episomal vector in which the SFFV promoter drives high-level transgene expression in hematopoietic cells and Woodchuck Hepatitis Virus Posttranscriptional Regulatory Element (WPRE) stabilizes transcribed mRNA, thereby increasing expression of transgenes. [40] This EV vector also led to successful generation of integration-free iPSCs from CB, even with OS alone. Here we used the same EV backbone to express several factors for reprogramming of PBMNCs. Our intent was to generate integration-free iPSCs from non-lymphoid PBMNCs, thus we cultured cells in conditions that favor expansion of HSCs and myeloid cells. After nucleofection of cultured PBMNCs with EV plasmids, cells were transferred to 6-well plates, pre-coated with feeder cells, for 3–4 weeks of culture. In contrast to CB, OS-expressing EV plasmid alone failed to reprogram PBMNCs, while inclusion of BCL-XL led to successful reprogramming (**Figure 2A**). A similar enhancing effect of BCL-XL was observed in OSK or OSMK-mediated reprogramming: BCL-XL increased reprogramming efficiency by up to 10-fold (**Figure 2B**). With the use of three EV plasmids expressing OS, MK and B (BCL-XL), up to 10 iPSC colonies could be generated (**Figure 2B**). Of interest, in the absence of MYC, OS+K+B also induced PBMNCs to pluripotency at a similar efficiency (**Figure 2B**).

To test whether the generated iPSCs are from non-lymphoid cells in PBMNCs, we nucleofected fractionated cells with EV plasmids expressing OS+MK+B. iPSCs were generated from the population of cells expressing the myeloid lineage marker, CD33, but not from the CD33^−^ cells (**Figure 2C**). To provide further evidence that iPSCs were indeed generated from non-lymphoid cells, we fractionated PBMNCs using antibodies that recognize T cells (CD3) and B cells (CD19). Consistent with our findings in the previous experiment, the T/B cell enriched population (containing CD3^+^ T and 19^+^ B lymphocytes, respectively) failed to generate any iPSCs, while the same number of T/B-depleted cells (CD3^−^/19^−^ cells) generated dozens of iPSCs (**Figure 2C**). Taken together, these data demonstrate that the integration-free iPSCs generated with our approach are derived from non-lymphoid cells.

**Figure 3:**
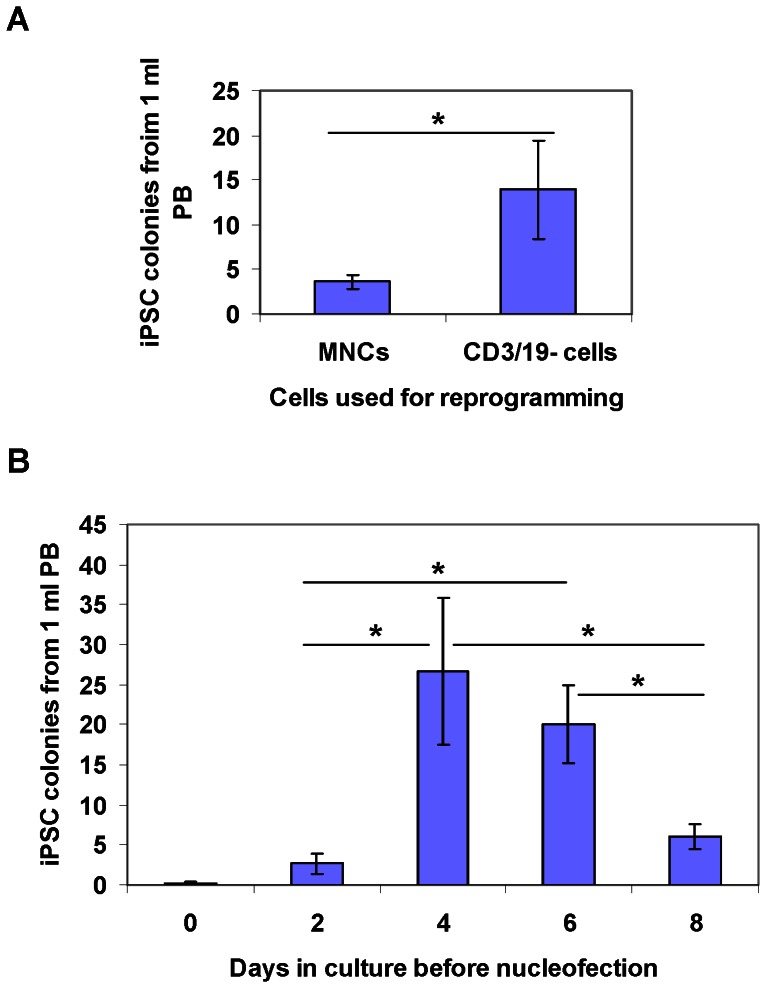
Optimization of the PBMNC reprogramming protocol. (**A**) Depletion of CD3^+^ and 19^+^ lymphoid cells increases reprogramming efficiency. Whole PBMNCs or CD3^−^/CD19^−^ cells (T/B cell-depleted PBMNCs) were cultured for 4 days before nucleofection with episomal vectors expressing OS (OCT4 and SOX2), MK (MYC and KLF4) and B (BCL-XL). 1×10^6^ cells were used for nucleofection. The numbers of iPSC colonies were counted at 3–4 weeks after nucleofection and numbers of iPSC colonies per 1 ml of PB were calculated by normalization to the amount of starting peripheral blood. Data shown are presented as mean ± SEM (n = 4). * indicates *P*<0.05. (**B**) Culturing PB CD3/19^−^ cells for 4 days allows maximum reprogramming. CD3/19^−^ cells (T/B cells-depleted) PBMNCs were cultured for 0 to 8 days before nucleofection with episomal vectors expressing OS, MK and B. 1×10^6^ cells were used for nucleofection. The numbers of iPSC colonies were counted at 3–4 weeks after nucleofection. Graphed data are presented as mean ± SEM (n = 6). * indicates *P*<0.05.

An incidental finding in the above experiments showed that T/B-depleted cells generated substantially more iPSC colonies (**Figure 2C**). We thus used PBMNCs from 4 donors to test whether T/B cell depletion can increase reprogramming efficiency. Whole PBMNCs or CD3^−^/19^−^ cells were cultured in the same conditions for 4 days and then nucleofected with EV plasmids expressing OS+MK+B. Approximately 10-fold more iPSC colonies were generated from 1×10^6^ CD3^−^/19^−^ cells than from 1×10^6^ whole MNCs. Since the amount of CD3^−^/19^−^ cells was only ∼30% that of MNCs after purification, this population generated iPSCs ∼3-fold more efficiently than when they were plated as unfractionated PBMNCs with T and B cells present (*P* < 0.05) (**Figure 3A**). In our pilot studies, we found that freshly isolated PBMNCs often failed to be reprogrammed and, after 10 days of *ex vivo* culture, the yield of iPSCs also substantially decreased. We then used CD3^−^/19^−^ cells to determine the optimal culture duration for efficient reprogramming. We found that if cells were cultured for less than 2 days, only a few colonies were obtained, whereas 4–6 days of culture led to the generation of more than 20 iPSC colonies from 1 ml of PB (**Figure 3B**). Culturing cells for more than 8 days led to a significant decrease in reprogramming efficiency (**Figure 3B**). Take together, T/B cell depleted PBMNCs that are cultured for 4–6 days under conditions that favor nonlymphoid cell expansion can be efficiently reprogrammed to iPSCs with EV plasmids that express five factors (OS+MK+BCL-XL).

### Characterization of integration-free iPSCs derived from adult PB

**Figure 4:**
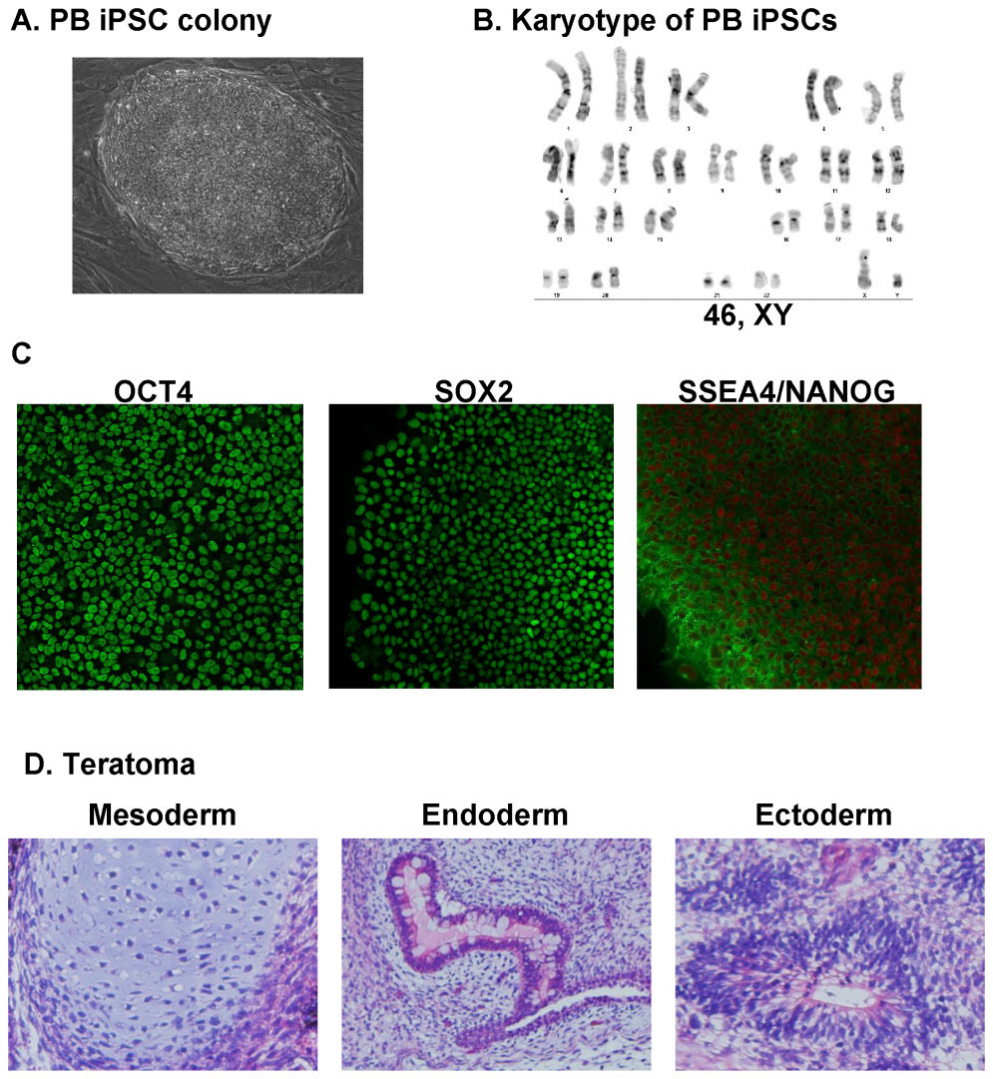
Characterization of integration-free PB iPSCs. (**A**) PB iPSCs show a typical morphology of human pluripotent stem cells. (**B**) A representative karyogram of an iPSC clone. All analyzed PB iPSC clones showed a normal karyotype. (**C**) PB iPSCs express pluripotency markers OCT4, SOX2, NANOG and SSEA4. Shown are representative confocal images captured using the Zeiss LSM 710 confocal microscope with a 20× objective. (**D**) PB iPSCs form teratoma in immunodeficient mice. H & E staining of representative teratoma from PB iPSCs shows derivatives of 3 embryonic germ layers. Cartilage (mesoderm); glands (endoderm) and neurotubules (ectoderm). Images were acquired using the Olympus microscope with a 20× objective.

PB iPSCs, generated as described above, robustly proliferated under human iPSC culture conditions for more than 20 passages. PB iPSC colonies showed a tight morphology characteristic of human pluripotent stem cells (**Figure 4A**). Consistent with earlier reports, [35], [40], [55] qPCR analysis of iPSCs after 10 passages showed that the average copy number of residual EV plasmids decreased to less than 0.01 copy per cell in 6 out of 6 iPSC clones, suggesting that after long-term culture, EV plasmids are depleted from almost all cells. Karyotype analysis indicated a normal human karyotype for all of the clones tested; one representative karyotype is shown in **Figure 4B**. Immunostaining of iPSC colonies showed expression of pluripotency markers like OCT4, SOX2, NANOG and SSEA4 (**Figure 4C**). Two months after being subcutaneously injected into immunodeficient mice, iPSCs formed teratomas consisting of derivatives of all three embryonic germ layers (**Figure 4D**). Together, these data demonstrate that integration-free PB iPSCs are morphologically, phenotypically and functionally identical to pluripotent stem cells.

### In vitro multilineage differentiation potential of integration-free PB iPSCs

**Figure 5:**
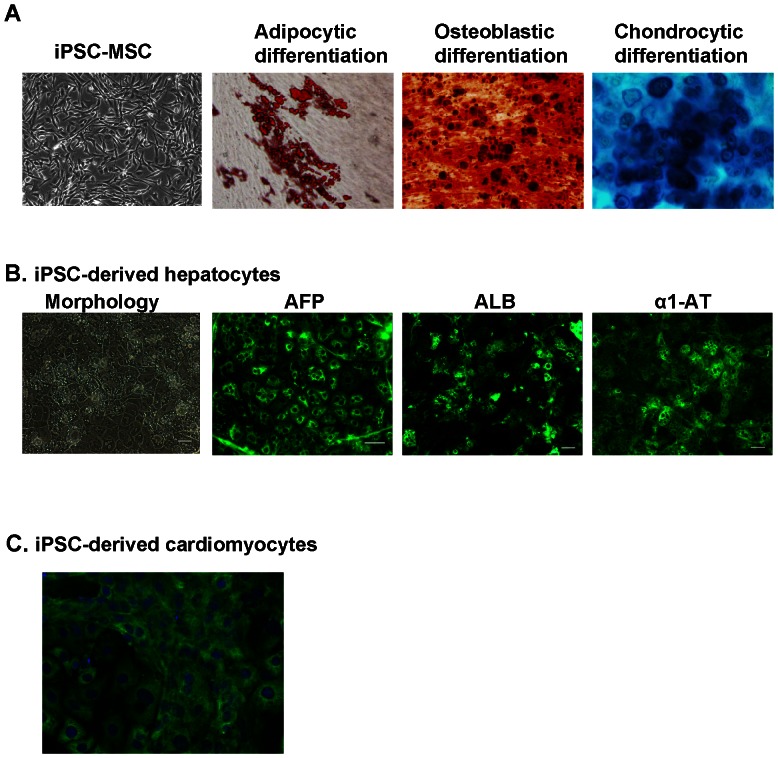
In vitro multilineage differentiation of integration-free PB iPSCs. (**A**) Differentiation of PB iPSCs into MSCs. iPSC-MSCs show a typical MSC morphology and are capable of differentiation into adipocytes, osteoblasts and chondrocytes. Oil Red O stains the oil droplets of adipocytes. Alizarin Red stains the bone nodules formed by osteoblasts. Alcian Blue stains acid mucopolysaccharides synthesized and secreted by chondrocytes. (**B**) PB iPSC-derived hepatocytes show a typical morphology of hepatocytes at 25 days after hepatocytic differentiation. These cells also express markers AFP, albumin (ALB), and alpha 1-antitrypsin (α1-AT). The differentiated cells were stained with monoclonal antibody against AFP, goat anti-albumin, and goat anti-alpha 1-antitrypsin at 18 days after differentiation culture. (**C**) PB iPSC can be induced to differentiation into cardiomyocytes that express Troponin I marker. Cell nuclei were counterstained with DAPI.

We further investigated whether PB iPSCs can be induced to differentiate into cells of different lineages in culture. We found that PB iPSCs were readily differentiated into mesenchymal stem cells (MSCs) in MSC-conducive culture conditions (**Figure 5A**). More than 90% cells expressed typical markers of MSCs including CD73, CD105 and CD166. Furthermore, after 3 weeks of induction culture, PB iPSC-derived MSCs differentiated into adipocytes, osteoblasts and chondrocytes (**Figure 5A**). These data suggest that MSCs differentiated from integration-free PB iPSCs are morphologically and functionally indistinguishable to bone marrow-derived MSCs. [56] To differentiate PB iPSCs into hepatocytes, cells were initially induced to a relatively homogenous population of definitive endoderm cells, which were further differentiated into hepatocyte-like cells within 7 days. The differentiating cells underwent a series of morphological changes. After Day 7, the cells manifested a polygonal shape and round single or double nuclei with many cytoplasmic vesicles, characteristic features of mature hepatocytes (**Figure 5B**). Immunohistochemical analysis showed that ∼90% cells iPSC-derived hepatocytes expressed liver-specific genes like alpha fetoprotein (AFP), albumin (ALB), and alpha 1-antitrypsin (α1-AT) (**Figure 5B**), thus providing evidence of *bona fide* differentiation into hepatocytes. We also investigated the cardiac differentiation capacity of PB iPSCs. After 2 weeks of culture in cardiomyocyte differentiation medium, dozens of beating colonies were observed in each well of 6-well plates. Immunostaining of these cells showed the majority of cells expressed the Troponin I marker, confirming their identity as cardiomyocytes (**Figure 5C**). Taken together, these in vitro differentiation data demonstrate that integration-free PB iPSCs can be induced to differentiate into MSCs, hepatocytes and cardiomyocytes.

## Discussion

Here we report that integration-free iPSCs can be efficiently generated from human adult PBMNCs in 3–4 weeks with EV plasmids that express five factors, OS+MK+BCL-XL. In previous studies we improved vector design to increase reprogramming efficiency of CB CD34^+^ cells by 10–20 fold. [40] Here we build on this improved vector design and show that inclusion of BCL-XL in the factor combination increases reprogramming efficiency by ∼10-fold. When we also enrich PBMNCs for non-lymphoid cells (CD3^−^CD19^−^) and combine this with *ex vivo* culture for 4–6 days, we can generate 20–30 iPSC colonies from 1 ml of PB. Consistent with our previous report on CB reprogramming, [40] we found that lentiviral vector-mediated transduction with OS alone can also induce PBMNCs into pluripotency, while inclusion of MK increases the reprogramming efficiency to a level that is more than 200-fold higher than previously reported. [46] Thus, our improved vector design provided a primary tool that with the inclusion of BCl-XL allows us to achieve unprecedented efficiency in PBMNC reprogramming. The improved vector design and the identification of BCL-XL as a powerful reprogramming-enhancing factor have important implications for cellular reprogramming of hematopoietic cells.

The reprogramming into pluripotency of unmobilized adult PBMNCs cells that lack the DNA alterations present in T and B lymphocytes (non-lymphoid cells) has been a long-sought goal. Recent technological breakthroughs make it possible to generate integration-free iPSCs from non-lymphoid cells in PB cells, but at relatively low efficiencies. Studies showed that 6–7 factors are necessary to achieve successful reprogramming of PBMNCs. In one report, the use of 6 factors (OSMK+LIN28+SV40 Large T antigen or SV40LT) leads to generation of ∼1 iPSC colony per ml of PB. [41] In others, the use of 7 factors (OSK+LIN28+NANOG+MYCL1+SV40LT or OSK+LIN28+MYCL1+TP53 shRNA+EBNA1) leads to the generation of 5–10 iPSC colonies from 1 ml of PB. [39], [42] In comparison, with only 5 factors (OS+MK+BCL-XL), we have achieved significantly higher reprogramming efficiency: 20–30 iPSC colonies from 1 ml of PB. With the inclusion of additional factors such as LIN28, Mir-302, TP53 shRNA and/or EBNA1, it is possible that we might generate ∼100 iPSCs colonies from 1 ml of PB, an efficiency that is 10–100 fold higher than previously reported. [39], [41], [42] The success is largely due to the use of the SFFV promoter in our vectors, which drives higher levels of transgene expression in hematopoietic cells than promoters like EF1 and PGK. Of interest, when only 4 factors (OS+KLF4+BCL-XL) were used, no obvious change in reprogramming efficiency was observed compared to conditions with MYC (**Figure 2B**). In some applications, MYC may pose potential risks and thus MYCL1 has been proposed to replace MYC. [38], [42], [57] Here we show that neither MYC nor MYCL1 are necessary for reprogramming PBMNCs, potentially broadening the applications of our approach.

Our studies demonstrate that the addition of BCL-XL increases reprogramming efficiency by ∼10-fold in all the three factor combinations we tested (**Figure 2B**); it is tempting to speculate that BCL-XL can also substantially enhance other factor combination-mediated reprogramming. This observation can be explained by three facts. First, the anti-apoptotic effect of BCL-XL may have led to better survival of cells that ectopically expressed reprogramming factors after transfection with EV plasmids. Second, a recent study shows that overexpression of BCL-XL in human embryonic stem cells (ESCs) not only attenuates apoptosis, but also upregulates the expression of adhesion molecules, which facilitate cell-matrix interactions. [58] The latter may be particularly important for the reprogramming of nonadherent PBMNCs, because tight adherence of PBMNCs to culture wells or matrix is the first step of successful reprogramming. Third, genomic analysis of human ESCs after long-term culture has identified recurrent amplifications at 20q11.21, a chromosome region that harbors the gene encoding BCL-XL. [59] This finding suggests that BCL-XL may also play a role in long-term self-renewal and proliferation of ESCs and iPSCs.

We found that integration-free PB iPSCs are reprogrammed from cells that express the CD33 myeloid marker, but not cells that express the T or B lymphocyte makers (CD3 or CD19, respectively). The culture conditions we used are favorable for the growth of HSCs and myeloid cells, but lack factors such as IL-2, IL-7 and anti-CD3 that support the growth and survival of lymphoid cells. In addition, nucleofection protocol we used is optimized for electroporation of nonlymphoid cells, in particular CD34^+^ cells. Thus, another benefit of our approach is that iPSCs are generated from non-lymphoid cells, which eliminates the need for screening of PB iPSCs to assure that they do not harbor genetic rearrangements at the TCR or IG gene.

The use of our lentiviral vector to express the four Yamanaka factors leads to the generation of more than 200-fold more iPSC colonies compared to one early study that used another vector backbone to express the same factors. [46] This lentiviral vector is likely to have broad application in the direct reprogramming of CB or PBMNCs into cells of clinical importance. Indeed, we recently found that SFFV-mediated expression of OCT4 directly reprograms blood CD34^+^ cells into induced MSCs, whereas the use of EF1 or PGK promoter failed to do so. [56] This result highlights the importance of choosing appropriate promoters when using lentiviral vectors to express genes in question.

Generation of integration-free iPSCs without the use of integrating viral vectors represents one step closer to the clinical application of iPSC-based therapy. However, there are other safety concerns of iPSCs, including epigenetic memory of starting cells, genetic mutation during reprogramming and immunogenicity of iPSC-derived cells. Epigenetic memory and aberrant epigenomic reprogramming have been identified in iPSCs. [60], [61] However, long-term passage of iPSCs has been found to be able to diminish epigenetic signature inherited from the parent cells. [62] Furthermore, inclusion of ascorbic acid in the culture media and the induction of high-level expression of Oct4 and Klf4 are able to prevent aberrant epigenetic variation, allowing for the generation of “all-iPSC mice. [63], [64] Early studies also showed high-level genetic aberrations in iPSCs, [65], [66], [67] but recent studies indicate that these identified mutations are largely due to fixation of preexistent rare mutations in the parental cells. [68], [69] Our exome sequencing analysis showed only 1.3 coding mutations per CB iPSC, suggesting that *de novo* mutations during CB reprogramming are negligible. [54] The report of immunogenicity of iPSCs even after syngeneic transplantation revealed another “dark side” of iPSCs. [70] However, comprehensive investigations into this issue by two labs could not reproduce that finding and instead demonstrated negligible immunogenicity of differentiated cells from iPSCs. [71], [72] Taken together, although early reports sparked pessimism over the therapeutic potential of iPSCs, several recent studies demonstrated their safety.

In summary, we are able to generate 20–30 integration-free iPSCs from the non-lymphoid cells in 1 ml of PB using EV plasmids expressing 4–5 factors including BCL-XL. To the best of our knowledge, this is the most efficient approach for generating iPSCs from PB with non-viral vectors thus far. In addition, the lentiviral vectors and EV plasmids reported here are likely to have other applications like direct reprogramming of blood MNCs cells into other types of cells for clinical therapy.

## Materials and Methods

### Cord blood and peripheral blood

The use of CB and PB was approved by the Institutional Review Board of Loma Linda University and written informed consent was obtained from all participants. CB CD34^+^ cells were purified by MACS as described previously. [40] MNCs were obtained by standard density gradient centrifugation with Ficoll-Hypaque (1.077 g/ml) at room temperature. Some PBMNCs were directly purchased from AllCells (Emeryville, CA). The age of male and female PB donors ranged from 22 to 43 years old. In some studies, cells enriched for the myeloid lineage marker, CD33, or T/B depleted (CD3^−^/CD19^−^) cells were used. CD33^+^ cells were purified by staining with CD33-PE antibody (eBioscience; San Diego, CA) followed by staining with PE Microbead Kit (Miltenyi Biotec, Auburn, CA). CD3^−^/CD19^−^ cells were collected from flow-through cells after being stained with CD3-PE and CD19-FITC (eBioscience) followed by isolation using anti-PE and FITC Microbead Kits (Miltenyi).

### Lentiviral and episomal vectors

Human BCL2, BCL-XL, and MCL1 cDNAs were purchased from Thermo Scientific Open Biosystems (Huntsville, AL). Open reading frames (ORFs) of these genes were cloned into lentiviral vector Lenti SFFV-OS under the control of the SFFV promoter as detailed previously. [40], [54] The backbone of this vector was modified from a lentiviral vector originally designed by Dr. Luigi Naldini. [73] The oriP/EBNA1-based episomal vectors EV SFFV-OS, EV SFFV-KLF4 and EV SFFV-MK have been described previously. [40] To drive expression of 2 genes, a self-cleavage peptide sequence from equine rhinitis A virus (E2A) was used to link the 2 genes. [9], [40] EV SFFV-BCL-XL was cloned by inserting BCL-XL into the EV backbone. All the constructs were verified by DNA sequencing. Lentiviral vector packaging and titering have been detailed elsewhere. [74] Biological titers of 5–10×10^7^/ml were routinely achieved in our lab after a 100-fold concentration by centrifugation at 6000 g for 24 hr at 4°C. [40], [74]

### Generation of iPSCs using lentiviral vector

For generation of iPSCs from CB, we followed our previously published protocol. [40], [54] To generate PB iPSCs, human PBMNCs were cultured in HSC culture conditions. [75], [76] Iscove's modified Dulbecco's medium (IMDM)/10% FBS supplemented with TPO, SCF, FL and G-CSF each at 100 ng/ml, IL-3 at 10 ng/ml, and StemRegenin1 or SR1 (Cellagen Technology; San Diego, CA) [77] at 1 uM. Cytokines were purchased from ProSpec (East Brunswick, NJ). After 6–8 days of culture, 1×10^5^ cells per well were seeded into non-TC treated 24-well plates that were pre-coated with fibronectin fragment RetroNectin or CH-296 (Takara Bio, Inc., Shiga, Japan). [78] Lentiviral transduction was conducted for 5–6 hr with a multiplicity of infection (MOI) of 4. One day after transduction, cells were harvested and transferred to 6-well plates, which were pre-seeded with inactivated rat embryonic fibroblast (REF) feeder cells (Applied Biological Materials Inc. or ABM; Richmond, BC, Canada). Cells were maintained in the HSC culture condition for 2 more days before being gradually replaced with iPSC medium. The iPSC medium is composed of Knockout DMEM/F12 medium (Invitrogen) supplemented with 20% Knockout Serum Replacement (KSR) (Invitrogen; Carlsbad, CA), 1 mM GlutaMAX (Invitrogen), 2 mM nonessential amino acids (ABM), 1× penicillin/streptomycin (ABM), 0.1 mM β-mercaptoethanol (Sigma-Aldrich Corp; St. Louis, MO), 20 ng/ml FGF2 (ABM), and 50 µg/ml ascorbic acid. [63], [79] Culture medium was changed every 2 days. To increase reprogramming efficiency, an inhibitor of histone deacetylase sodium butyrate [80], [81] was added at 0.25 mM every 2 days from day 2 to 10, and cells were cultured under hypoxia throughout the experiment by placing culture plates in a hypoxia chamber (Stemcell Technologies, inc., Vancouver, BC, Canada) that was flushed with mixed air composed of 92%N_2_/3%O_2_/5%CO_2_. [40], [82] [54]. Starting from day 10, REF-conditioned medium was used.

### Generation of integration-free iPSCs with episomal vectors

PBMNCs or fractionated cells (CD33^+^ or CD3^−^/CD19^−^) cells) were cultured under HSC conditions for 2–8 days. To generate integration-free iPSCs, cells were nucleofected with 15–20 µg EV plasmid DNA using human CD34 Cell Nucleofector® Kit (Lonza). In each nucleofection, 10 µg EV SFFV-OS, 5 µg EV SFFV-BCL-XL, 5 µg EV SFFV-KLF4 or 5 µg EV SFFV-MK were used. 1×10^6^ cells were nucleofected with Amaxa Nucleofector II using program U-008. Immediately after nucleofection, cells were cultured in CH-296 pretreated well plates. The next day, cells were transferred to each well of 6-well plates that were preseeded with REF. Cells were cultured the exactly same way as for reprogramming with lentiviral vector expect that every 7–10 days, 2–3×10^5^ freshly thawed inactivated REF feeder cells were added into each well. The number of ALP-positive iPSC colonies was counted at 3–4 weeks after nucleofection. Some iPSCs colonies were picked for further culture. iPSCs were passaged every 5–7 days by treating with Dispase (Invitrogen). After 10–20 passages, iPSCs were further characterized with other assays.

### ALP staining

To determine the number of iPSC colonies, culture plates were stained with an ALP-staining kit (Stemgent, Inc. San Diego, CA). ALP-positive iPSC colonies were enumerated at 2–3 weeks after transduction for lenti iPSCs or 3–4 weeks after nucleofection for episomal iPSCs.

### Confocal imaging

For immunostaining of iPSC colonies, iPSCs were cultured in chamber slides for 4–5 days. Cells were treated with fixation buffer and permeabilization buffer (eBioscience) for 30 min before being stained overnight at 4°C with PE or FITC conjugated antibodies anti-OCT4 (eBioscience), anti-SOX2 (BD Pharmingen; San Diego, CA), anti-NANOG (BD Pharmingen), and anti-SSEA-4 (eBioscience). Confocal imaging was performed using the Zeiss LSM 710 NLO laser scanning confocal microscope with a 20× objective at the Loma Linda University Advanced Imaging and Microscopy Core. High resolution monochrome images were captured using a Zeiss HRm CCD camera.

### Teratoma assay

The use of NOD/SCID/IL2RG^−/−^ (NSG) immunodeficient mice for the teratoma formation assay was approved by the Institutional Animal Care and Use Committee at Loma Linda University (LLU). NSG mice were purchased from the Jackson Laboratory (Sacramento, CA) and maintained at the LLU animal facility. Approximately 1×10^6^ iPSCs were suspended in 200 ul DMEM/F12 diluted (1∶1) Matrigel solution (BD) and injected into the subcutaneous tissue above the rear haunch of NSG mice. At 2 months after implantation, teratomas were dissected and fixed in 10% formalin. After microsectioning, samples were stained with hematoxylin and eosin (H & E) and analyzed by a board certified pathologist.

### Karyotyping and G-banding

GTG-banding chromosome analysis was carried out in the LLU Radiation Research Laboratories. Standard DNA spectral karyotyping procedures were followed and a HiSKY Complete Cytogenetic System was used (Applied Spectral Imaging, Inc. Vista, CA). For each clone, 10 metaphases were analyzed and karyotyped.

### In vitro differentiation of integration-free PB iPSCs

To differentiate PB iPSCs into MSCs, iPSCs were cultured with Mesenchymal Stem Cell (MSC) Medium Kit (ABM) for 4–5 days. Cells were then treated with Accutase (Innovative Cell Technologies, Inc., San Diego, CA) and further cultured in fibronectin (BD)-precoated non-tissue culture treated well plates. After 5 passages under MSC culture conditions, cells were induced to differentiate into adipocytes, osteoblasts, and chondrocytes as detailed previously. [56] After 3 weeks of culture in differentiation medium, Oil Red O, Alizarin Red, and Alcian Blue staining for adipocytes, osteoblasts, and chondrocytes, respectively, were conducted. [56]

To differentiate iPSCs into cardiomyocytes, small clusters of iPSCs were cultured in differentiation medium consisting of StemPro-34 (Invitrogen), supplemented with 2 mM GlutaMAX, 50 µg/ml ascorbic acid, and 4×10^−4^ M monothioglycerol (MTG) (Sigma). [83] Cytokine Activin A (R &D Systems; Minneapolis, MN) was used at 50 ng/ml for one day and 10 ng/ml BMP4 (R&D Systems) was added for four days. After 12 days of culture, beating colonies of cardiomyocytes were observed. The identity of cardiomyocytes was confirmed by immunostaining with cardiomyocyte-specific marker Troponin I (R&D Systems).

Hepatic differentiation of iPSCs was performed as previously described. [84] Briefly, induction of iPSC towards definitive endoderm (DE) was initiated in RPMI 1640 medium containing 100 ng/ml Activin A (R&D Systems) and 2 mM L-glutamine for 2 days. B27 (Invitrogen) and 0.5 mM sodium butyrate were then supplemented for additional 7–9 days. The DE cells were treated with Accutase for a short period of time and rapidly transferred to collagen I-coated plates and cultured in RPMI 1640 medium supplemented with FGF-4, HGF, BMP2, BMP4 (R&D Systems Inc.), dexamethasone, and DMSO as previously described. [84] Fourteen days after differentiation induction, the cells were maintained in hepatocyte culture medium supplemented with FGF-4, HGF, Oncostatin M (R&D Systems Inc), dexamethasone, and DMSO for an additional 2–3 weeks. At 18 days after differentiation, cells were stained with monoclonal antibody against AFP (Dako; Carpinteria, CA), goat anti-albumin (Bethyl Laboratories; Montgomery, TX), and goat anti-alpha 1-antitrypsin (Bethyl), following standard protocols.

### Statistical analysis

Data are presented as mean ± standard error of the mean (SEM). Two-tailed Student *t* test was performed. *P* values of <0.05 were considered statistically significant.
